# Supplementary material for: Deleterious Mutations in the TPO Gene Associated with Familial Thyroid Follicular Cell Carcinoma in Dutch German Longhaired Pointers
Source: Genes (Basel). 2021 Jun 29;12(7):997. doi: 10.3390/genes12070997 (PMC8306087; doi:10.3390/genes12070997)
Supplement: Supplementary file 1 [file genes-12-00997-s001.zip › genes-1193315/Supplementary_figures.pdf]

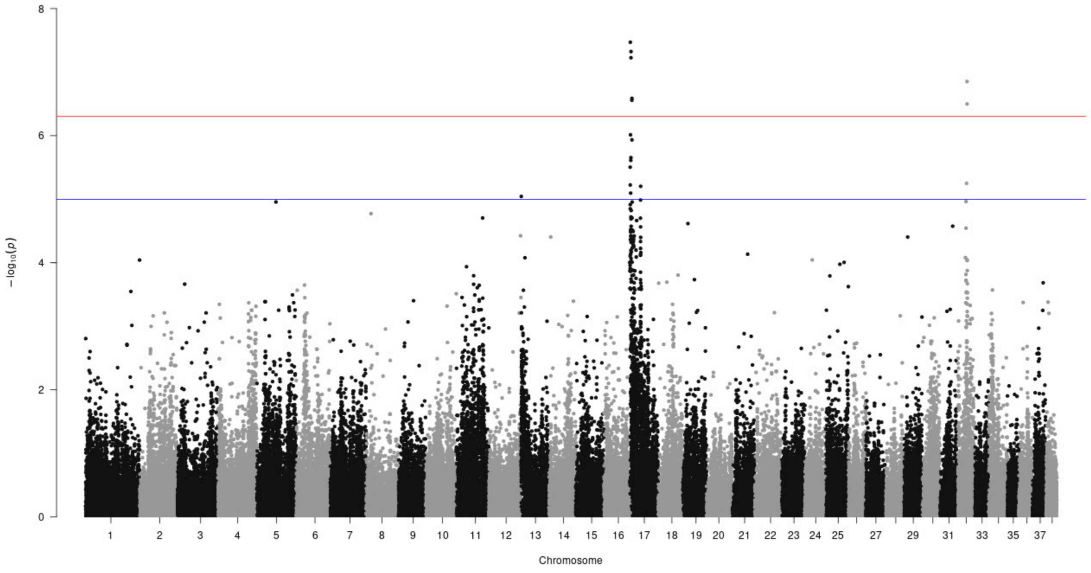

Supplementary Figure S1. Manhattan plot of GWAS result across the whole genome.

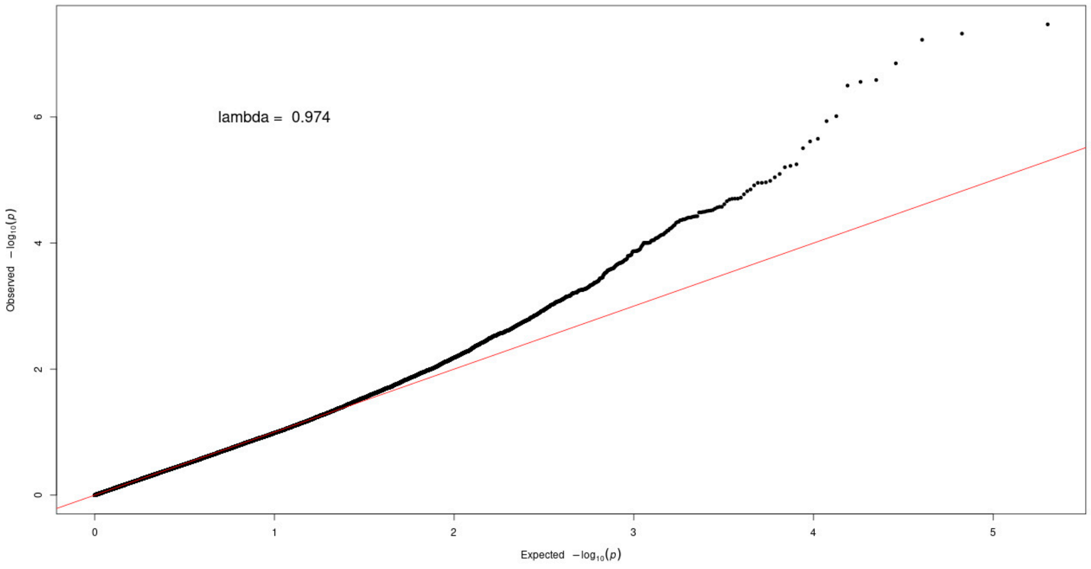

Supplementary Figure S2. QQ plot of the GWAS result. Inflation value is 0.974.

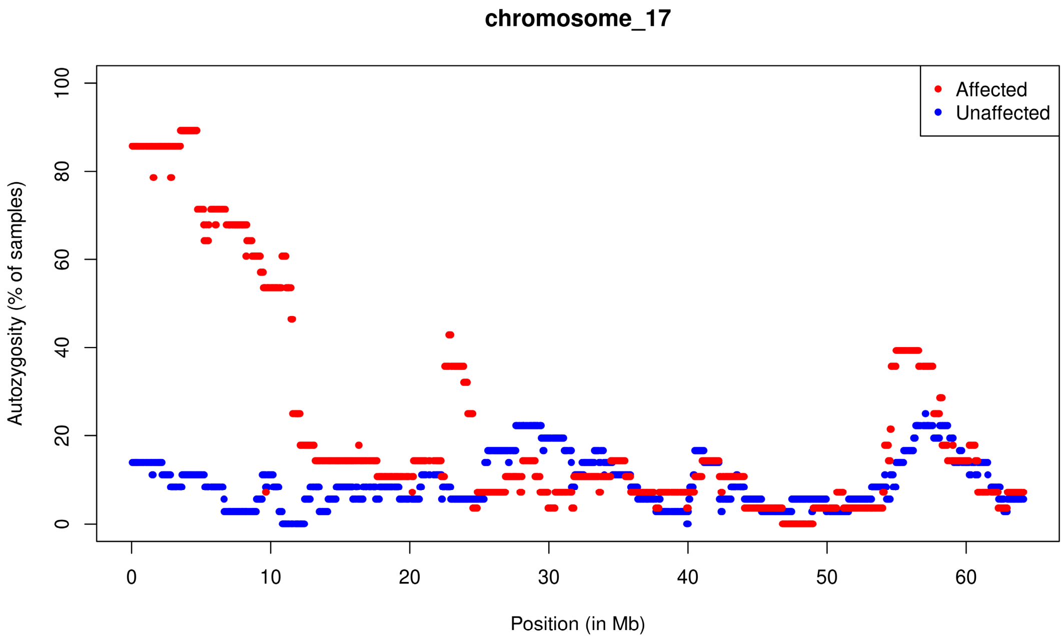

Supplementary Figure S3. Autozygosity of ROH segments on chromosome 17 between the affected and unaffected GLPs.

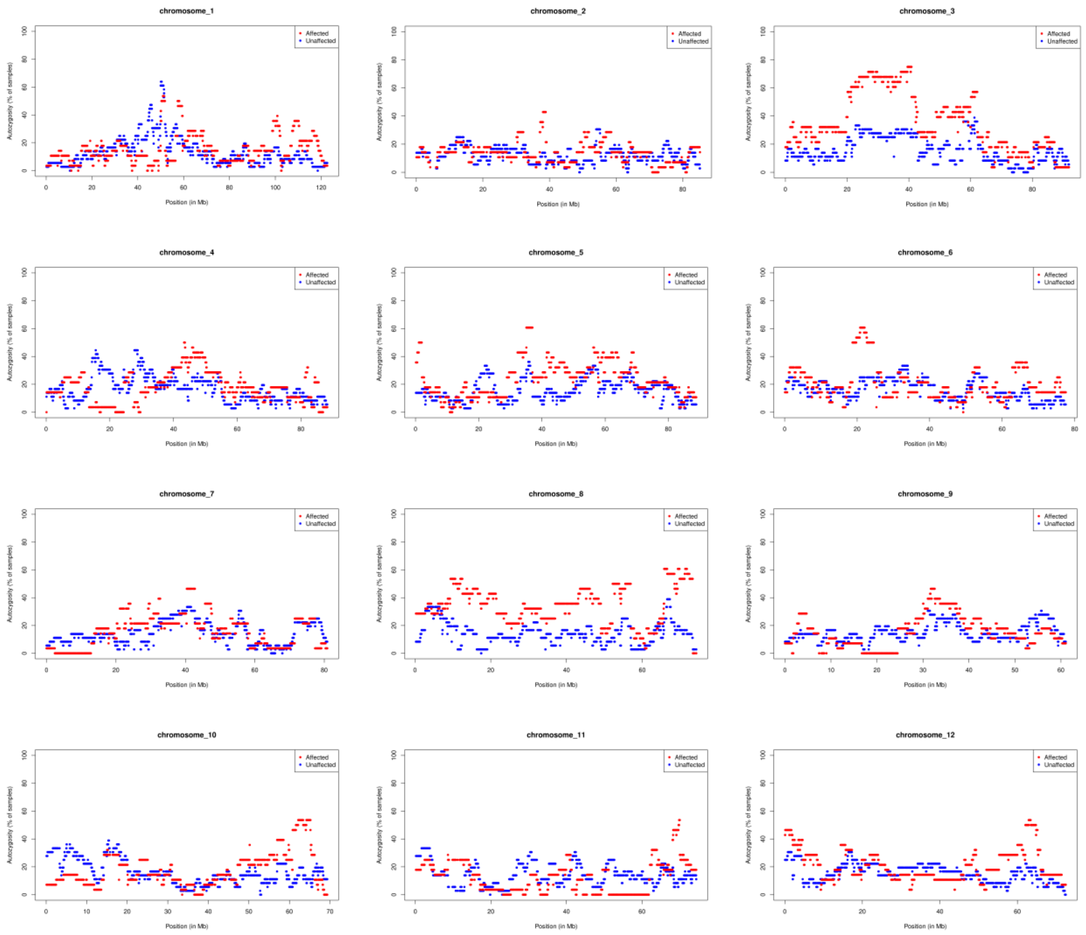

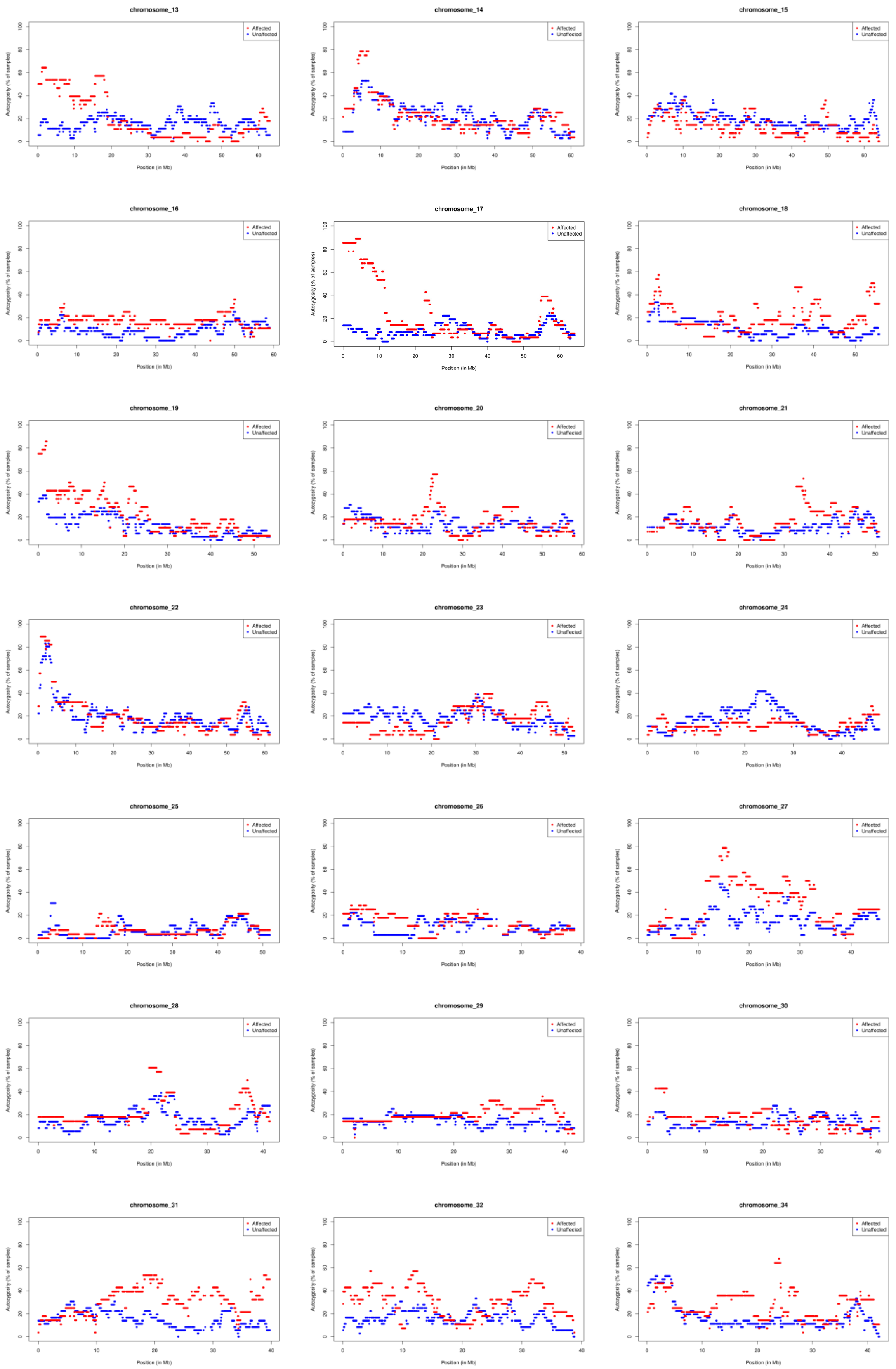
chromosome_13
chromosome_14
chromosome_15
chromosome_16
chromosome_17
chromosome_18
chromosome_19
chromosome_20
chromosome_21
chromosome_22
chromosome_23
chromosome_24
chromosome_25
chromosome_26
chromosome_27
chromosome_28
chromosome_29
chromosome_30
chromosome_31
chromosome_32
chromosome_34
Affected
Unaffected
Autozygosity (% of samples)
Position (in Mb)

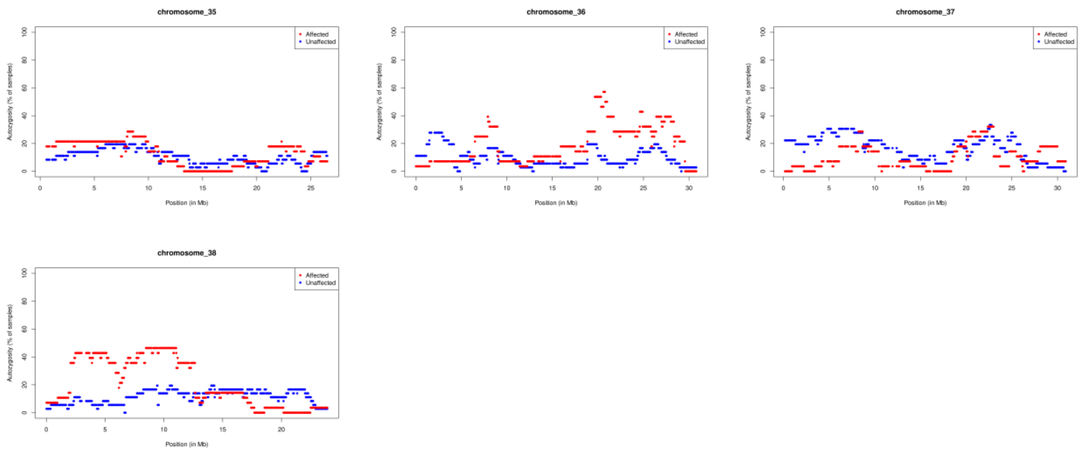

Supplementary Figure S4. Autozygosity of ROH segments in affected and unaffected GLPs on each autosomal chromosome.

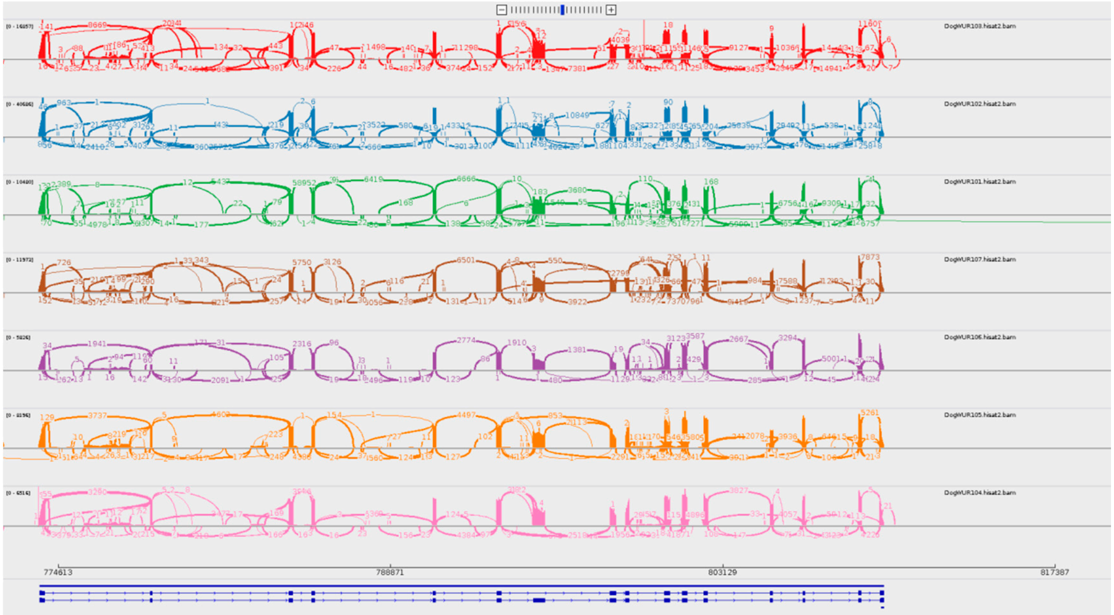

Supplementary Figure S5. Sashimi plot of the TPO mRNA.

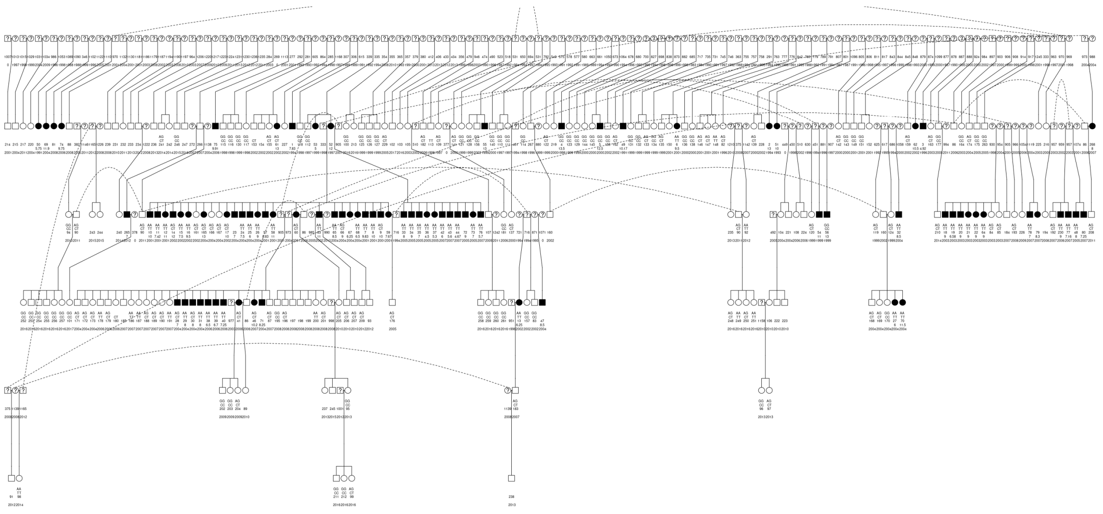

Supplementary Figure S6. Pedigree of dogs genotyped. Dog in black color were affected. A question mark denotes the unknown affected status. Five rows of label below the circle or square are genotype of chr17:800788G>A, genotype of chr17:805276C>T, ID of dog, diagnoses age, and year of birth, respectively.

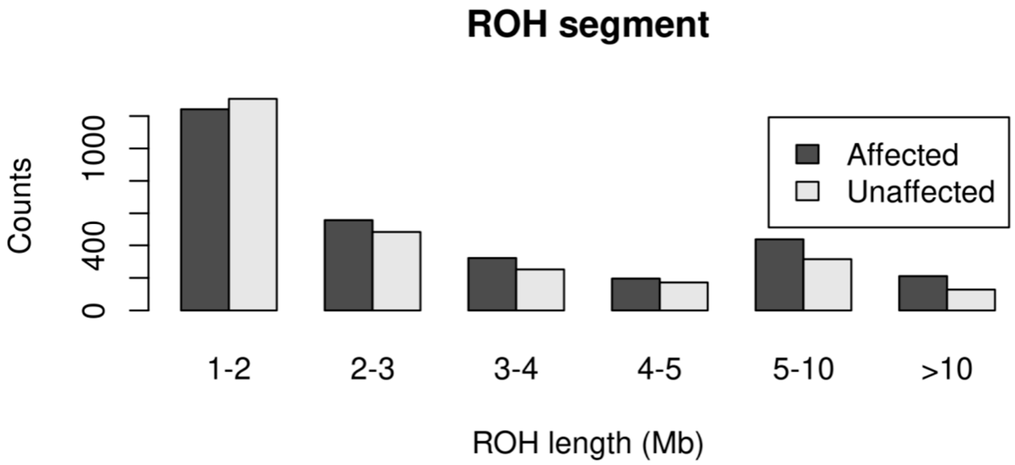

Supplementary Figure S7. The Counts of ROH segments based on SNP array genotype data in different lengths between affected and unaffected GLPs. Affected dogs have more ROH segments with length above 2 Mb.

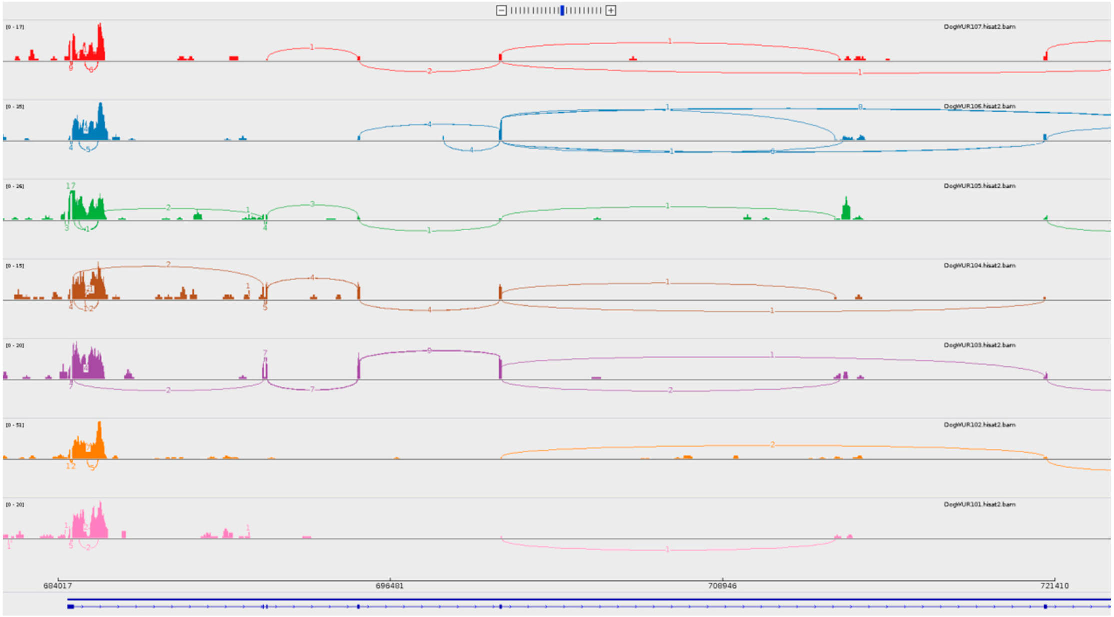

Supplementary Figure S8. Sashimi plot of the first 6 exons of SNTG2 mRNA.

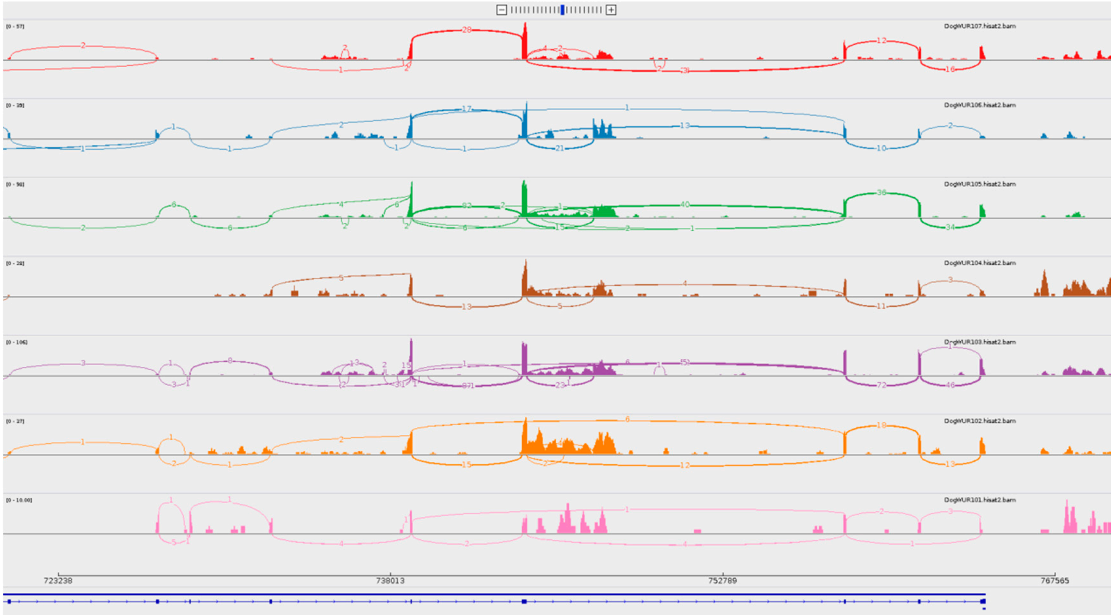

Supplementary Figure S9. Sashimi plot of the last 9 exons of SNTG2 mRNA.
